# Breast imaging in patients with nipple discharge

**DOI:** 10.1590/0100-3984.2016.0103

**Published:** 2017

**Authors:** Ivie Braga de Paula, Adriene Moraes Campos

**Affiliations:** 1 MSc, Member of the Colégio Brasileiro de Radiologia e Diagnóstico por Imagem (CBR), MD, Radiologist at Conrad Diagnóstico por Imagem, Belo Horizonte, MG, Brazil.; 2 Member of the Colégio Brasileiro de Radiologia e Diagnóstico por Imagem (CBR), MD, Radiologist at Conrad Diagnóstico por Imagem, Belo Horizonte, MG, Brazil.

**Keywords:** Nipple discharge, Mammography, Ultrasonography, Magnetic resonance imaging, Derrame papilar, Mamografia, Ultrassonografia, Ressonância magnética

## Abstract

Nipple discharge is a common symptom in clinical practice, representing the third leading breast complaint, after pain and lumps. It is usually limited and has a benign etiology. The risk of malignancy is higher when the discharge is uniductal, unilateral, spontaneous, persistent, bloody, or serous, as well as when it is accompanied by a breast mass. The most common causes of pathologic nipple discharge are papilloma and ductal ectasia. However, there is a 5% risk of malignancy, mainly ductal carcinoma in situ. The clinical examination is an essential part of the patient evaluation, allowing benign nipple discharge to be distinguished from suspicious nipple discharge, which calls for imaging. Mammography and ultrasound should be used together as first-line imaging methods. However, mammography has low sensitivity in cases of nipple discharge, because, typically, the lesions are small, are retroareolar, and contain no calcifications. Because the reported sensitivity and specificity of ultrasound, it is important to use the correct technique to search for intraductal lesions in the retroareolar region. Recent studies recommend the use of magnetic resonance imaging in cases of suspicious nipple discharge in which the mammography and ultrasound findings are normal. The most common magnetic resonance imaging finding is non-mass enhancement. Surgery is no longer the only solution for patients with suspicious nipple discharge, because short-time follow-up can be safely proposed.

## Abstract (pt)

O derrame papilar é um sintoma frequente na prática clínica, correspondendo à terceira queixa mais comum, sendo precedido apenas por dor e massas palpáveis. A maioria dos derrames papilares é de origem benigna e transitória, sendo definidos como derrames papilares patológicos os que se apresentam uni ou pauciorificiais, espontâneos, persistentes, serosos ou sanguinolentos e associados a alteração palpável. Os derrames patológicos são mais frequentemente causados por papiloma ou ectasia ductal, porém, existe risco de malignidade de cerca de 5%, constituído principalmente por carcinoma ductal *in situ*. O exame clínico é parte essencial na avaliação da paciente, permitindo diferenciar entre derrames papilares tipicamente benignos e derrames papilares suspeitos, que necessitam de avaliação pelos métodos de imagem. A mamografia e a ultrassonografia devem ser usadas em conjunto como métodos de imagem de primeira linha, porém, a sensibilidade da mamografia nestes casos é baixa, uma vez que as lesões são comumente retroareolares, pequenas e não calcificadas. A sensibilidade e a especificidade da ultrassonografia variam amplamente na literatura, sendo importante o uso de técnicas corretas para a avaliação de lesões intraductais e retroareolares. Recentemente, a ressonância magnética tem sido indicada nos casos de derrame papilar suspeito com mamografia e ultrassonografia normais, sendo o achado mais comum o realce não nodular. A cirurgia não é mais a única solução para as pacientes com derrame papilar suspeito e todos os exames de imagem normais, tendo em vista que um seguimento em curto prazo pode ser proposto de forma segura.

## INTRODUCTION

Nipple discharge is quite common, with a prevalence of 5-10%, representing the third leading breast complaint, after pain and lumps ^(1,2)^. It is considered suspicious when it occurs spontaneously and is persistent, unilateral, bloody, or serous, as well as when it occurs in patients who are not pregnant or breastfeeding. In most cases, suspicious nipple discharge is caused by benign lesions such as ductal ectasia, in 6-59% of cases, and papilloma, in 35-56% ^(3)^. The risk of underlying malignancy is not negligible, ranging from 5% to 23% ^(2)^.

Anamnesis and physical examination, with visual inspection and palpation of the breasts and papillae, play essential roles in the differentiation between physiological and pathological nipple discharge. The approximate date of onset of the symptom should be investigated, as should its duration, frequency, and quantity, as well as whether it is spontaneous. It is also important to investigate the date of the last pregnancy, recent breastfeeding, use of medications (anticoagulants or neuroleptics), trauma, and smoking, as well as patient hormonal status and (personal and family) history of breast or ovarian disease. The visual inspection should ideally be made with the aid of a lamp or loupe, which allows nipple discharge to be distinguished from false nipple discharge, which derives from lesions of the nipple-areola complex. The nipple discharge should be defined as uniductal or multiductal and as unilateral or bilateral. The color of the liquid should be evaluated, which is best done by placing a little of it onto a piece of gauze.

Physiological (i.e., non-suspicious) nipple discharge has the following characteristics: bilateral; non-spontaneous; previous or intermittent; multiductal; and milky, green or dark in color. In contrast, nipple discharge that is unilateral, spontaneous, persistent, serous, or bloody should be considered pathological and should be investigated by imaging.

The color of the secretion determines whether cytology analysis is necessary. Although cytology has the advantage of being easy to perform and painless, it has the disadvantage of variable sensitivity, with a > 50% rate of false-negative results for malignant lesions ^(4)^. For the cytological examination of the material from the nipple surface, the secretion can be placed on a dry slide (if Giemsa staining is used) or on a slide fixed in ethanol (if Papanicolau staining is used).

Nipple discharge in men should always be considered a suspicious finding, because the incidence of carcinoma in this context is approximately 23% ^(5)^. It occurs in 25% of cases of invasive ductal carcinoma, and axillary lymph node enlargement is common at the time of diagnosis. Suspicious calcifications occur in 13-30% of cases ^(6)^.

Imaging methods play a fundamental role in the assessment of patients with nipple discharge and make it possible to perform precise imaging-guided biopsies, which provide tissue specimens to be analyzed by the pathologist. At most facilities, if papilloma is identified in the biopsy specimen, surgical excision is performed, because papilloma can be associated with carcinoma ^(7)^. Recent studies show that, in cases of papilloma that is single, intraductal, central, and small, diagnosed by vacuum-assisted breast biopsy and presenting no cellular atypia in the pathological examination, clinical follow-up and imaging can preclude the need for surgery ^(8,9)^.

## IMAGING METHODS FOR THE ASSESSMENT OF NIPPLE DISCHARGE

### Mammography

Mammography plays an important role in the diagnosis of breast diseases ^(10-15)^. Although mammography should always be the first examination requested, it has low (20-25%) sensitivity in cases of nipple discharge ^(16)^, because the associated lesions are usually retroareolar, small, intraductal, and noncalcified ^(17)^. Therefore, negative mammography results do not exclude the possibility of underlying disease.

**Figure 1:**
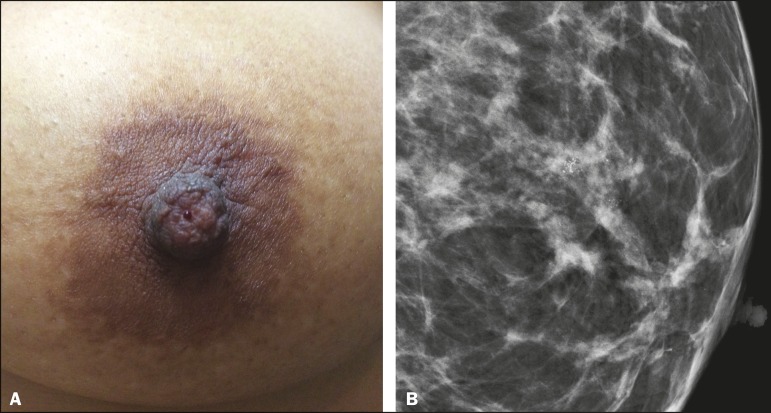
**A:** Photograph of the nipple-areola complex in a patient with grade II DCIS that is solid, cribriform, and necrotic, with unilateral bloody nipple discharge. **B:** Magnified mediolateral oblique view showing fine pleomorphic calcifications with segmental distribution in the retroareolar region of the left breast.

The main mammography finding is calcification. The calcifications are typically benign, including eggshell calcifications, which can be associated with papilloma, and rod-shaped calcifications, which are usually associated with ductal ectasia. There can also be calcifications of suspicious morphology and distribution, such as pleomorphic calcifications and calcifications with a segmental or linear distribution ^(1)^, as depicted in Figure 1. Mammography can also reveal nodules, focal asymmetry, and ductal ectasia.

In cases of nipple discharge, more attention should be paid to the retroareolar region. There are no protocols in the literature for specific analysis of that region during mammography. However, when there is suspicion, localized compression or magnification should be used.

### Ultrasound

Ultrasound should always be performed in cases of nipple discharge, even if the alteration has already been noted on mammography ^(5)^. Bahl et al. ^(17)^ found that, for the detection of ductal carcinoma *in situ* (DCIS) or invasive carcinoma in patients with suspicious nipple discharge, the sensitivity and specificity of ultrasound were 56% and 75%, respectively.

Appropriate technique includes use of high-frequency transducers, heated gel and ambient temperature control to avoid contraction of the musculature of the nipple and areola. To improve the visualization of the nipple and subareolar regions, certain maneuvers, such as tilting the transducer and observing along the axis of the duct, with discrete peripheral compression, should be used ^(18)^.

One of the main ultrasound findings is ductal ectasia, defined as a duct caliber greater than 3 mm. In patients with suspicious nipple discharge who show focal ductal ectasia with anechoic content, the lesion should be biopsied, because that finding is seen in half of all cases of papilloma and in 14% of all cases of DCIS ^(1,19)^. Focal ductal ectasia in a peripheral location, irregular duct margins, thickening of the duct wall, and hypoechoic adjacent tissue are characteristics that can indicate malignancy ^(20)^.

In the presence of pathological nipple discharge, subareolar nodules and acoustic shadowing should be classified as BI-RADS 4 or 5 findings. Such findings can be related to DCIS, which is difficult to diagnose by ultrasound, because false-negative results are obtained in approximately 80% of cases ^(1)^.

**Figure 2:**
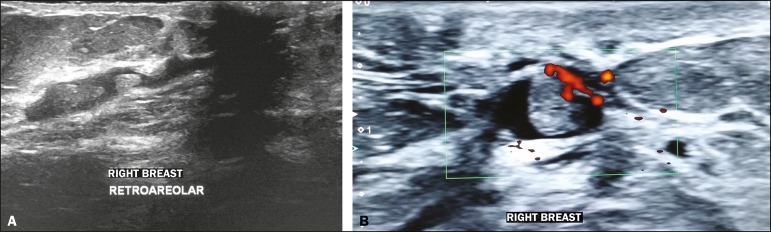
**A:** Ultrasound showing intraductal nodules. **B:** Doppler ultrasound showing vascularity within an intraductal nodule.

Doppler ultrasound can facilitate the differentiation between a duct producing viscous secretions and an intraductal nodule, because it can reveal vascularization within the latter ^(17)^. The most common cause of an intraductal nodule is a single papilloma located a few centimeters from the nipple, usually resulting in ductal obstruction (Figure 2). The characteristics that increase the risk of malignancy are being over 50 years of age, presenting with a nodule larger than 1 cm, and the nodule being located more than 3 cm from the nipple ^(20)^.

**Figure 3:**
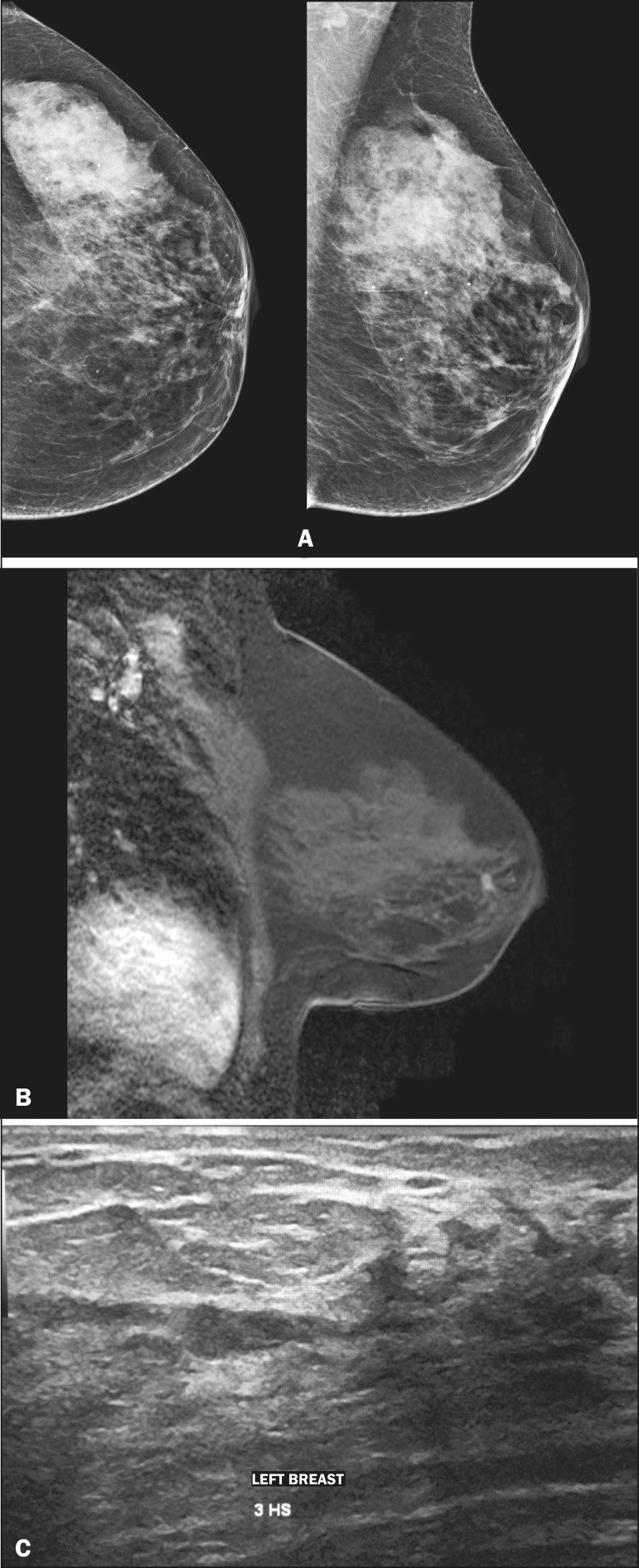
A 64-year-old patient with bloody discharge from the left nipple. **A:** Mammography in craniocaudal and mediolateral oblique views, showing focal asymmetry in the retroareolar region. **B:** T1-weighted MRI sequence with fat suppression, 2 min after intravenous injection of gadolinium, showing a nodule with ill-defined margins at the same location. **C:** Second-look ultrasound showing a hypoechoic intraductal nodule, in correspondence with the mammography and MRI findings. Evaluation of a biopsy specimen demonstrated intraductal papilloma without atypia.

Ultrasound is important in the second-look evaluation after magnetic resonance imaging (MRI) and can be used to guide biopsies or to facilitate the preoperative wire-guided localization. Ultrasound is better at detecting nodules than non-mass lesions ^(21)^, as can be seen in Figure 3.

### MRI

**Figure 4:**
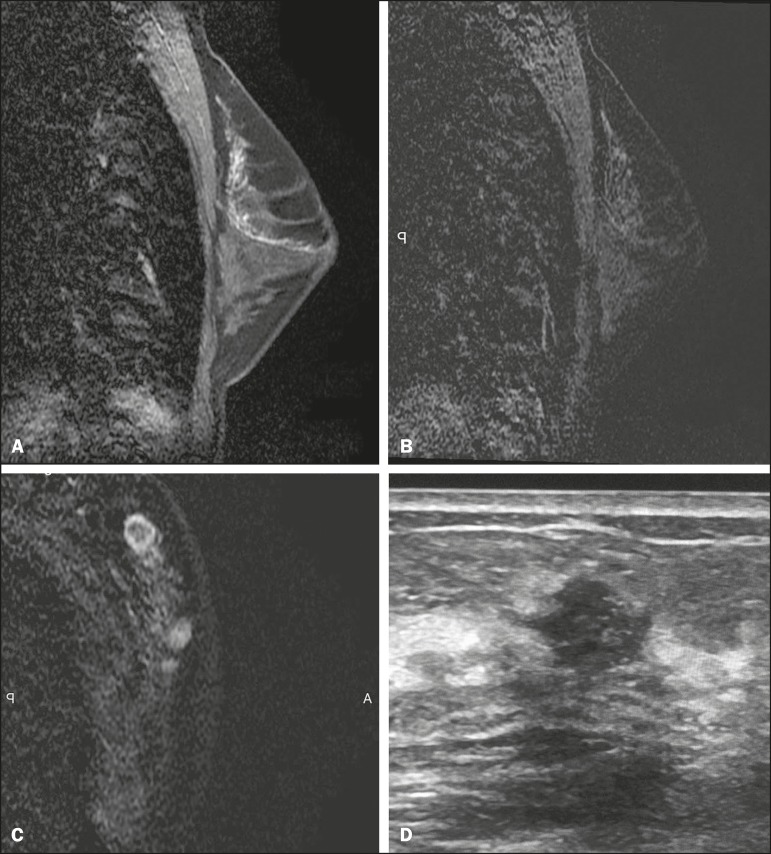
A 44-year-old patient with suspicious nipple discharge. Mammography showing dense breasts with focal asymmetry in the superolateral quadrant of the left breast. Ultrasound, obtained at another facility, showing no alterations. **A:** Sagittal T1-weighted MRI sequence with fat suppression, showing ductal ectasia with hemorrhagic and high protein content in the superolateral quadrant of the left breast. **B:** MRI with digital subtraction 2 min after intravenous administration of contrast medium, showing non-mass enhancement with segmental distribution and heterogeneous enhancement in the superolateral quadrant of the left breast. **C:** Contrast-enhanced MRI with digital subtraction, showing a nodule with ill-defined margins and ring enhancement in the superolateral quadrant of the left breast, in correspondence with the mammography finding. **D:** Second-look ultrasound showing a nodule with ill-defined margins in the superolateral quadrant of the left breast, in correspondence with the MRI findings. Evaluation of a biopsy specimen demonstrated grade II invasive mucinous carcinoma.

There have been few studies on the use of MRI in cases of nipple discharge. According to the European Society of Breast Cancer Specialists, nipple discharge is an emerging indication that has yet to be validated, the evidence produced in the studies warranting only a Grade C recommendation. In clinical practice, MRI can be performed in patients with suspicious nipple discharge in whom mammography and ultrasound findings have been normal ^(22)^. The negative predictive value of MRI is good (approximately 90%), low-grade or very small DCIS lesions accounting for the false-negative results ^(22-24)^. In the assessment of the location and extent of a lesion, MRI is superior to mammography and ultrasound ^(1,25)^. In addition, MRI can identify lesions that initially went unnoticed but could be seen on the second-look ultrasound or mammography, especially lesions occurring in the retroareolar region (Figure 4).

The main MRI finding in patients with suspicious nipple discharge is non-mass enhancement. In a study of 47 patients with suspicious nipple discharge, 59% of the malignant lesions showed non-mass enhancement with segmental distribution, 57% showed heterogeneous enhancement within the lesion and 40% showed a plateau-type enhancement curve ^(26)^. In T1-weighted sequences, high protein or hemorrhagic content within the duct can appear as an area of high signal intensity, simulating linear or segmental enhancement. In order to differentiate between the two findings, the pre-contrast and digital subtraction sequences must be evaluated. In the presence of nipple discharge, a focus of contrast enhancement should be considered suspicious, because it could represent a papilloma.

The main criticisms of MRI are its high cost, the detection of additional alterations that can call for other follow-up tests or biopsies unrelated to the initial clinical complaint, and the difficulty of determining whether the lesion is intraductal or not ^(25)^. For that purpose, a second-look ultrasound examination is indispensable.

### Galactography

Galactography, also known as ductography, has long been considered the gold standard for the evaluation of nipple discharge. A study by Manganaro et al. evaluated 53 patients with unilateral nipple discharge who underwent galactography and MRI, comparing the two methods in terms of their ability to identify diseases and to distinguish between benign and malignant lesions. In the identification of ductal disease, MRI showed higher sensitivity than did galactography (98% vs. 49%) and both methods presented high specificity. Unlike galactography, MRI was able to demonstrate not only ductal disease but also lesions in the adjacent parenchyma ^(27)^.

## DISCUSSION

Although MRI plays an increasingly greater role in the study of breast cancer ^(28,29)^, there have been few studies on its use in cases of nipple discharge.

Despite the lack of reliable scientific evidence of the benefit of using MRI in patients with suspicious nipple discharge in whom mammography and ultrasound findings are normal, most authors recommend performing MRI of the breasts. If the MRI scan identifies a suspicious lesion, it is now routine practice to use a second-look ultrasound to localize the finding. However, if MRI shows non-mass enhancement with linear or segmental distribution, corresponding to the site of nipple discharge, second-look mammography with magnification of the region can be useful in the investigation of suspicious calcifications, allowing stereotactic biopsy to be performed. If no abnormality is found, an MRI-guided biopsy of the suspicious lesion should be performed ^(1)^.

Historically, surgical resection of the terminal breast ducts was the rule for patients with suspicious nipple discharge in whom mammography, ultrasound, and MRI all produced normal results. It has recently been shown that the risk of developing a malignant lesion is quite low in such patients, especially if there are no other suspicious clinical signs. In addition, when such patients do develop a malignant lesion, it is a low-grade DCIS or a very small tumor. Therefore, the most recent studies in the literature recommend that patients with suspicious nipple discharge in whom mammography, ultrasound, and MRI findings are all normal should be followed for two years, with follow-up evaluations every 6 months, until there is spontaneous resolution of the discharge, which occurs in 81% of the cases ^(1,16,30)^. The follow-up protocol can be ultrasound and clinical examinations every 6 months, together with annual mammography. However, for patients with massive nipple discharge, nipple discharge that causes discomfort, or nipple discharge that persists for more than two years, surgery should be considered ^(1)^.

## FINAL CONSIDERATIONS

The majority of cases of suspicious nipple discharge have a benign cause, the risk of malignancy being approximately 5% and DCIS accounting for most such malignancies. After clinical evaluation and physical examination, the imaging investigation begins with mammography and ultrasound, with special attention to the retroareolar region. In such cases, mammography has a sensitivity of 20-25% for the detection of suspicious lesions, compared with 65-85% for ultrasound. When the mammography and ultrasound findings are normal, MRI can be used, because it has high sensitivity for lesions of the nipple and malignant lesions. The most common MRI finding is non-mass enhancement, being more suspicious for malignancy when presenting segmental distribution and heterogeneous internal enhancement. When the MRI findings are suspicious, second-look mammography or ultrasound can facilitate the biopsy process. For patients in whom all imaging examinations produce normal results, a follow-up protocol involving clinical examination, mammography, and ultrasound can be suggested, given that spontaneous resolution of nipple discharge occurs in a large number of cases.
